# Some Personal Advice Concerning How to Write Precise, Concise and Eloquent Research Articles

**DOI:** 10.3389/fspor.2021.648929

**Published:** 2021-03-18

**Authors:** Hans-Christer Holmberg, Billy Sperlich

**Affiliations:** ^1^Department of Physiology and Pharmacology, Biomedicum C5, Karolinska Institutet, Stockholm, Sweden; ^2^School of Kinesiology, University of British Columbia, Vancouver, BC, Canada; ^3^Integrative and Experimental Exercise Science, Department of Sport Science, University of Würzburg, Würzburg, Germany

**Keywords:** article writing, optimizing research presentation, creating a scientific manuscript, writing science, abstract, effective scientific writing, organizing your scientific article, writing research articles for maximal impact

## Introduction

Apparently, many young scientists (and some more experienced ones, as well) do not fully understand the importance of crafting scientific manuscripts that are both clear and enhance the impact of their message. Consequently, insufficient time and effort are often devoted to manuscripts prior to submission for publication. Here, we make some suggestions that can hopefully be of value in this context.

## The Different Sections of the Manuscript

### The Title

Naturally, you want other scientists to read your article and the title is your major form of advertising. Make it as specific and concrete as possible. If a conclusion can be stated simply, do so and indicate its significance. At the same time, do these things as concisely as possible, since this might possibly exert some impact on how often the paper is cited (Letchford et al., 2015).

### The Abstract

Briefly state the **BACKGROUND**, i.e., what gap in our knowledge are you attempting to fill and why it is important to do so. The **AIMS** will then follow logically. Describe the principles underlying your **METHODOLOGICAL** approach, avoiding distracting minor details. This allows the reader to assess whether your approach is appropriate and reliable. The **RESULTS** should provide specific values, including statistical analyses, rather than merely writing that parameters “increased,” “decreased” or, even worse “were different.” After all, it matters whether something increases by 5 or 500%. The **CONCLUSIONS** should not simply restate the results, but instead describe the new knowledge obtained and propose future specific studies/practical applications. Do not simply write “more research is needed.”

The Abstract is a key element of your manuscript, so give this section extra tender loving care. It is important to be as accurate as possible (i.e., avoid false advertising) to attract the right audience. Include as much information as possible within the word limit specified by the journal.

*The Keywords* should help others find your work in databases. It is unnecessary to repeat keywords in the Title and/or Abstract.

### The Introduction

The Introduction should provide a brief description of your research question in several individual paragraphs comprised of short sentences and each containing a basic piece of information, concept or idea. Begin in general terms and then rapidly focus in on the key knowledge gap, making this so clear that readers could formulate the aims themselves. The information should be arranged in a manner that it culminates logically in your concluding hypotheses or research question(s). Aims have highest impact when stated as hypotheses tested; next highest when formulated as research question(s); and least impact if you simply state you did something to see what would happen. At the end of the Introduction, in some disciplines authors also summarize very briefly their methodological approach and/or conclusions. If you do this, keep it quite short.

### The Methods

Your peer reviewers will certainly focus on this section, which may be the single most important part of your manuscript. In our experience, methodology that is inappropriate, described in insufficient detail and/or not at least briefly motivated is one major reason for rejection of manuscripts. Other researchers must have enough information to attempt to verify or refute your findings, as well as to apply your approach to their own problems (In praise of replication studies and null results, 2020).

The Methods should describe the characteristics of your experimental and control subjects (age, sex, height, mass, nationality), their level of performance (descriptive – national/regional team – and/or based on the standards of an international body, e.g., Fédération Internationale de Ski (FIS) points), their specific sporting discipline (e.g., sprint cross-country skiers), relevant physiological parameters (e.g., VO_2max/peak_) and years/volume of training. Clearly, enough participants should be included to allow statistically relevant conclusions to be drawn. In this context, performing a power analysis in advance can be helpful (Statistical Power Analysis–Statistics Solutions, 2021). Indeed, performing a quantitative study with too little power to allow a statistically significant conclusion to be drawn can be ethically questionable.

As indicated above, the methodology, apparatus, and procedures employed should be described in sufficient detail to allow others to attempt to reproduce your results. For instance, the names of manufacturers and suppliers of the equipment utilized, including URLs where available, should be reported. Be aware that much published research in the life sciences has proven to be extremely difficult to reproduce (Begley and Ellis, 2012; Baker, 2016). The reasons for this vary, but include inadequate description of methodology.

The statistical procedures used to analyze the data should be described and, at least briefly, motivated. In this context, advice from an expert statistician will almost certainly be beneficial (Ioannidis, 2005). Some journals have now incorporated independent assessment of statistical analyses into the review process (Hardwicke and Goodman, 2020). To facilitate interpretation of your data, also include and discuss both experimental and biological variation.

All investigations involving humans and/or non-human animals should explicitly state that the protocols were pre-approved by an ethical review board or committee, i.e., that experimental animals were not subjected to any unnecessary discomfort, stress or pain. In addition, it should be stated explicitly that human subjects provided their informed written consent before participating.

We also suggest that tables and figures be used more often to clarify aspects of your methodology. For example, if you have a list of things in the text (e.g., antibodies, criteria for inclusion and exclusion, experimental conditions), ask yourself whether this information could be presented both more accessibly and in less space as a table. If your experimental procedure involves a number of sequential steps, it can be highly illustrative to present these as a flow diagram. You could include a photograph of your experimental set-up. In addition, figures can often help clarify descriptions of complicated procedures in the text.

### The Results

The data included in the main manuscript should be directly relevant to and focused on your research question and underlying mechanisms. Other relevant data which would disturb this focus can be presented as supplementary material. Admittedly, it is not always easy to distinguish between these two, but it is well worth making an effort to do so.

Present your findings mostly in tables and figures, which can convey a great deal of information (including statistics) concisely and allow readers to draw their own conclusions (Vigen, 2015). Each table and figure should be comprehensible on its own, i.e., the legend should explain briefly the aim, methodology and statistical analysis involved. Unless your measurements are unusually accurate, values should not include more than three significant digits—sometimes fewer. Some helpful guidelines concerning the number of significant digits to include are available (Cole, 2015).

In general, tables and figures have higher “impact” than text. Since they are often the “heart” of your article, put at least as much time and effort into revising tables and figures as you do into revising the text. It is now very easy to make a variety of figures and tables, even in PowerPoint and Microsoft Word, as well as in a number of other, more powerful software programs. So, try formatting tables and figures in different ways, and compare these before deciding which to use.

**Figure 1:**
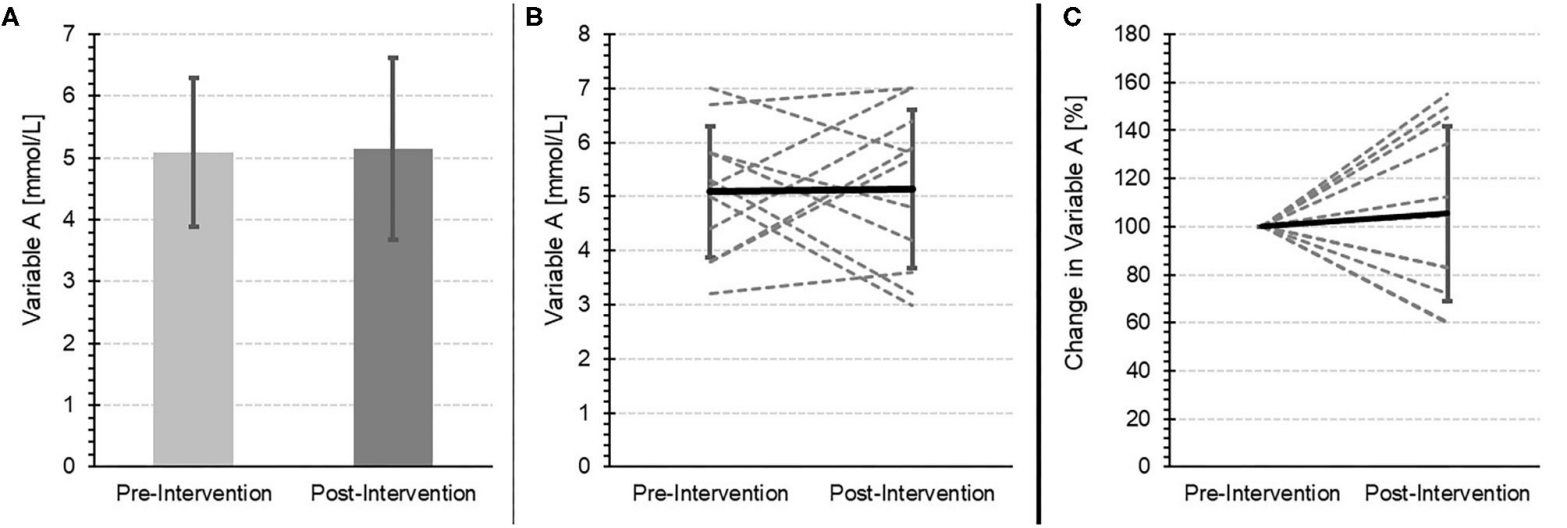
The same values for a fictitious variable measured before and after an intervention. **(A)** “Traditional” comparison of the means and standard deviations. **(B)** A Spaghetti-Plot with inter- and intra-individual (gray-dotted line), as well as mean (black line) changes. **(C)** Inter- and intra-individual (gray-dotted line), as well as mean (black line) percentage changes.

In exercise physiology, individual changes are often of considerable interest. Therefore, in addition to means and standard deviations, we urge researchers to present intraindividual changes, perhaps as a spaghetti plot (see Figure 1).

The text should describe what the author wants the reader to understand from a table or figure. In general, we believe that you should not repeat exact values provided in a figure or table in the text without a very good reason for doing so (e.g., emphasizing specific values).

Always remember that a statistically significant correlation does not necessarily indicate a causal relationship (Vigen, 2015; Do Storks Deliver Babies, 2021) and always provide the number of independent measurements (n). Calculate effect size and confidence intervals whenever appropriate. The cut-off value of *P* < 0.05, commonly used as an indication of statistical significance, is arbitrary. Rather, present actual *P*-values. Avoid discussing “tendencies” or “trends,” as many reviewers will object. Merely considering percentages or fold-changes can be misleading, so always consider the actual value of the parameter of interest as well.

Again, as mentioned above, if you have additional data that the reader should or might like to have access to, but which would distract from your main message, provide this in the Supplementary Material or Appendix. Remember, every unnecessary piece of information detracts from the impact of the important findings.

### The Discussion

Start by briefly summarizing your major findings, but without repeating exact data from the Results. This makes your novel information clear to peer reviewers and, later on, readers. It also forces you to decide which findings you should focus on in the Discussion.

Thereafter, discuss possible underlying mechanisms. Why did you get these results, what is happening? Mechanisms, particularly molecular mechanisms, have very high “impact” in the natural sciences.

Next, compare your findings to those of other relevant publications and attempt to explain any discrepancies. If your findings disagree with those of others in the area, compare their publication to your own manuscript in minute detail, looking for any differences (especially in methodology) that might explain the discrepancy.

Consider the possible limitations of your own study—paradoxically, most reviewers consider an awareness and openness about potential weaknesses as a strength. However, do not forget to emphasize strengths as well.

Discuss the possible consequences of your observations and/or future investigations required or motivated. Be as concrete as possible about future perspectives. As in the abstract, writing the equivalent of “more research is needed” is meaningless—more research is always needed. Describe the hypotheses, questions or mechanisms that need to be investigated and/or methods that should be applied, concretely and concisely. And, of course, if your research findings have potential practical implications, discuss these in some detail as well.

Finally, state your conclusions—have you supported or rejected the hypothesis you posed, or obtained an answer to your research question?

## General Discussion With Some Overriding Rules

Each journal has specific requirements regarding how to format and compose a manuscript, as well as other aspects of the submission process, usually including adherence to the international system of units (SI units). Please read and adhere to the Instructions to Authors carefully, in order to minimize the necessity for preliminary inspection by the editor and technical revision.

Use subheadings in all sections (even if the journal does not allow this; a word processor makes it easy to remove these later if necessary). Subheadings make the structure clear not only to the reader, but also to you, the author.

Use lots of figures and tables throughout the manuscript (this is rarely done in the Introduction and Discussion, be pioneers). These often present information in a more accessible manner than text.

Do not provide irrelevant or unnecessary information. This detracts seriously from the impact of your main message. Relevant but unnecessary information can be provided in the Supplementary Material.

Finally, pay attention to the language you use. Like it or not, the manner in which a message is received is heavily influenced by the manner in which the message is formulated. Write simply and directly. If at all possible, unless you are a native English speaker (and sometimes even if you are), let someone who is (and, preferably, is also a scientist) take a look at your manuscript before submitting it for publication. This will almost always shorten your text and remember, every unnecessary word detracts from the impact of the important words. In addition, the message should become easier to understand and more direct, with enhanced “impact” (as well as, hopefully, linguistically more varied and eloquent).

**Figure 2:**
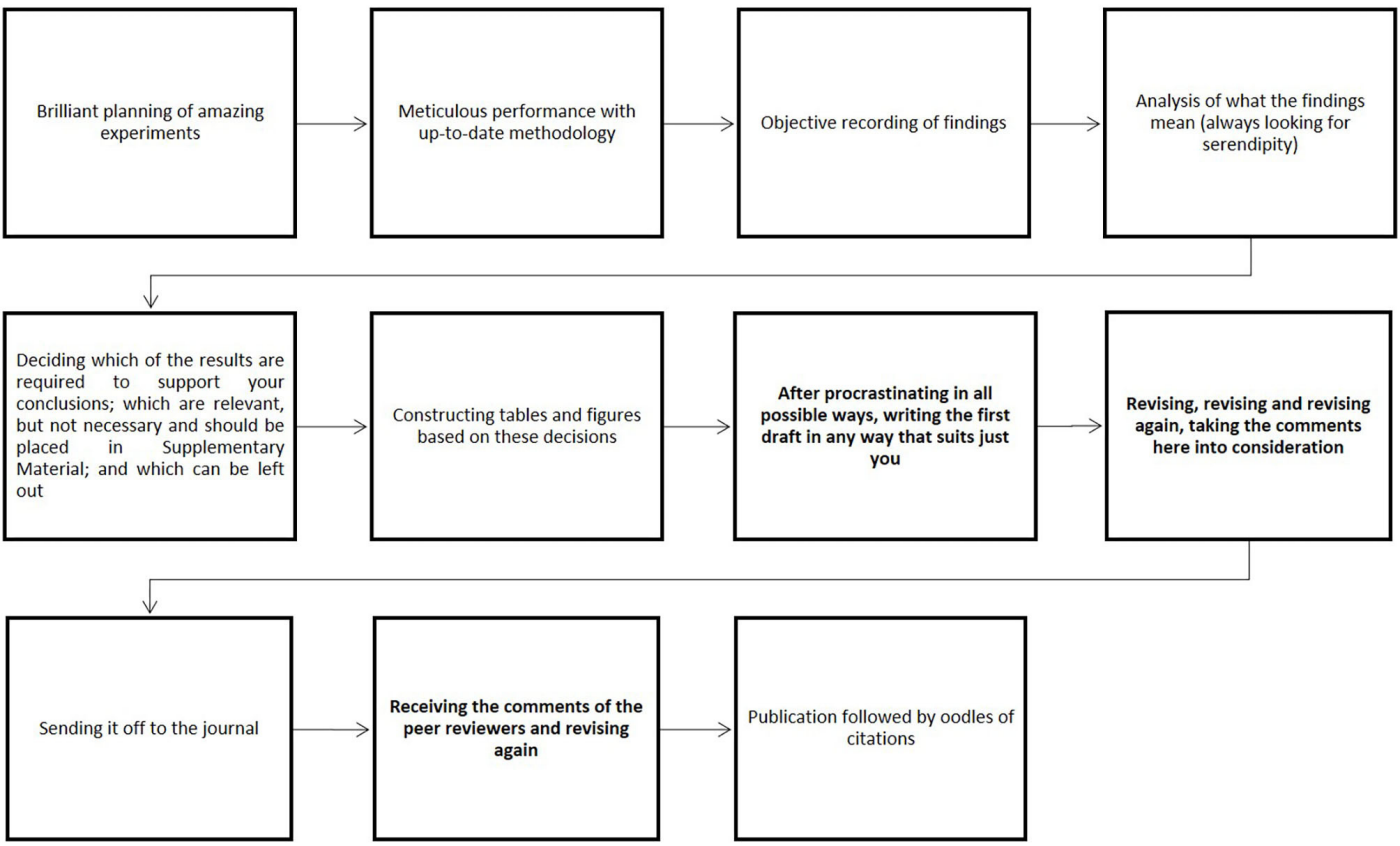
Schematic illustration of the creation of a scientific article.

For a variety of reasons, including being more interested in findings than in communicating them, many scientists put more effort into conducting interesting and important experiments than into communicating their results in an optimal fashion. Personally, we feel that senior scientists should not only mentor junior colleagues in how to plan, perform and interpret research, but also help them develop their skills in communicating research in both an effective and ethical manner (see Figure 2). We hope that the thoughts expressed here will be of value in this context.

## Author Contributions

H-CH: conceptualization and writing—original draft. H-CH and BS: writing—review and editing. Both authors have approved the final version of the manuscript and qualify for authorship.

## Conflict of Interest

The authors declare that the research was conducted in the absence of any commercial or financial relationships that could be construed as a potential conflict of interest.
